# Biomarkers of extracellular matrix formation are associated with acute-on-chronic liver failure

**DOI:** 10.1016/j.jhepr.2021.100355

**Published:** 2021-08-27

**Authors:** Annarein J.C. Kerbert, Saurabh Gupta, Eman Alabsawy, Iwona Dobler, Ida Lønsmann, Andrew Hall, Signe Holm Nielsen, Mette J. Nielsen, Henning Gronbaek, Àlex Amoros, Dave Yeung, Jane Macnaughtan, Rajeshwar P. Mookerjee, Stewart Macdonald, Fausto Andreola, Richard Moreau, Vicente Arroyo, Paolo Angeli, Diana J. Leeming, William Treem, Morten A. Karsdal, Rajiv Jalan

**Affiliations:** 1 Institute for Liver and Digestive Health, University College London, Royal Free Campus, London, UK; 2 Translational and Biomarker Research, GI-DDU, Takeda Pharmaceuticals International Co., Cambridge, MA, USA; 3 Statistical and Quantitative Sciences, Takeda Pharmaceuticals International Co., Cambridge, MA, USA; 4 Biomarkers and Research, Nordic Bioscience, Herlev, Denmark; 5 Sheila Sherlock Liver Centre, Royal Free London NHS Foundation Trust, London, UK; 6 Department of Biomedicine and Biotechnology, Technical University of Denmark, Lyngby, Denmark; 7 Department of Hepatology and Gastroenterology, Aarhus University Hospital, Aarhus, Denmark; 8 European Foundation for the Study of Chronic Liver Failure, Barcelona, Spain; 9 Inserm and Université de Paris, Centre de Recherche sur l’Inflammation (CRI), Paris, France; 10 Service d’Hépatologie, Hôpital Beaujon, Assistance Publique-Hôpitaux de Paris, Clichy, France; 11 Unit of Internal Medicine and Hepatology, Department of Medicine, DIMED, University of Padova, Padua, Italy; 12 Clinical Science, GI-TAU, Takeda Pharmaceuticals International Co., Cambridge, MA, USA; 13 Faculty of Medicine, Alexandria University, Alexandria, Egypt

**Keywords:** Liver cirrhosis, Multi-organ failure, Collagen, Prognosis, α-SMA, alpha-smooth muscle actin, ACLF, acute-on-chronic liver failure, AD, acute decompensation, cK18, caspase-cleaved keratin 18, CLIF-C ACLF, CLIF Consortium Acute-on-Chronic Liver, CLIF-C AD, CLIF Consortium Acute Decompensation, CLIF-C OF, CLIF Consortium Organ Failure, CPE, concordance probability estimate, DAMP, danger-associated molecular pattern, ECM, extracellular matrix, HC, healthy control, HR, hazard ratio, HSC, hepatic stellate cell, INR, international normalised ratio, IHC, immunohistochemistry, K18, keratin 18, MELD, model for end-stage liver disease, MMP, matrix metalloproteinase, NGAL, neutrophil gelatinase-associated lipocalin, NIS, noninterventional Study, PAMP, pathogen-associated molecular pattern, ROC, receiver operating characteristic, SC, stable cirrhosis, TLR, toll-like receptor, UCL, University College London, UCLH, University College London Hospitals, WCC, white cell count

## Abstract

**Background & Aims:**

Acute-on-chronic liver failure (ACLF) is characterised by organ failure(s), high short-term mortality, and, pathophysiologically, deranged inflammatory responses. The extracellular matrix (ECM) is critically involved in regulating the inflammatory response. This study aimed to determine alterations in biomarkers of ECM turnover in ACLF and their association with inflammation, organ failures, and mortality.

**Methods:**

We studied 283 patients with cirrhosis admitted for acute decompensation (AD) with or without ACLF, 64 patients with stable cirrhosis, and 30 healthy controls. A validation cohort (25 ACLF, 9 healthy controls) was included. Plasma PRO-C3, PRO-C4, PRO-C5, PRO-C6, and PRO-C8 (*i.e.* collagen type III–VI and VIII *formation*) and C4M and C6M (*i.e.* collagen type IV and VI *degradation*) were measured. Immunohistochemistry of PRO-C6 was performed on liver biopsies (AD [n = 7], ACLF [n = 5]). A competing-risk regression analysis was performed to explore the prognostic value of biomarkers of ECM turnover with 28- and 90-day mortality.

**Results:**

PRO-C3 and PRO-C6 were increased in ACLF compared to AD (*p* = 0.089 and *p* <0.001, respectively), whereas collagen degradation markers C4M and C6M were similar. Both PRO-C3 and PRO-C6 were strongly associated with liver function and inflammatory markers. Only PRO-C6 was associated with extrahepatic organ failures and 28- and 90-day mortality (hazard ratio [HR; on log-scale] 6.168, 95% CI 2.366–16.080, *p* <0.001, and 3.495, 95% CI 1.509–8.093, *p* = 0.003, respectively). These findings were consistent in the validation cohort. High PRO-C6 expression was observed in liver biopsies of patients with ACLF.

**Conclusions:**

This study shows, for the first time, evidence of severe net interstitial collagen deposition in ACLF and makes the novel observation of the association between PRO-C6 and (extrahepatic) organ failures and mortality. Further studies are needed to define the pathogenic significance of these observations.

**Lay summary:**

This study describes a disrupted turnover of collagen type III and VI in Acute-on-chronic liver failure (ACLF). Plasma biomarkers of these collagens (PRO-C3 and PRO-C6) are associated with the severity of liver dysfunction and inflammation. PRO-C6, also known as the hormone endotrophin, has also been found to be associated with multi-organ failure and prognosis in acute decompensation and ACLF.

## Highlights

• Collagen type III and VI formation is increased in ACLF compared to AD.
• PRO-C3 and PRO-C6 correlate with the severity of liver dysfunction and inflammation in AD and ACLF.
• High PRO-C6 levels were found to be indicative for the presence of multi-organ failure and worse survival.

## Graphical abstract

**Figure:**
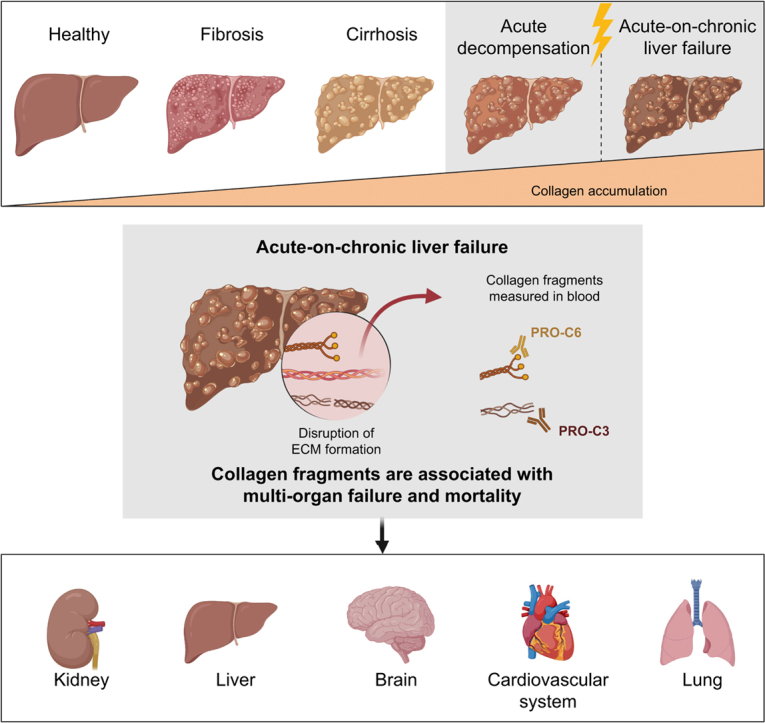

## Introduction

Liver fibrosis is a consequence of chronic hepatocyte injury and is characterised by excessive deposition of extracellular matrix (ECM) consisting of different collagen types that are produced by hepatic myofibroblasts derived from activated hepatic stellate cells (HSCs).[1], [2], [3] Fibrosis progression is a dynamic process that involves both formation and degradation of the ECM and that becomes unbalanced during liver disease. During this remodelling process, endopeptidases such as metalloproteinases (MMPs), can degrade collagen by cleavage, leading to the release of telopeptides in the circulation. The sites of degradation by specific MMPs are distinct, and the fragments, also known as neo-epitopes, serve as markers of tissue degradation quantified by collagen degradation.^4^ Similarly, the pro-peptides that are released during the maturation of the collagen molecule can serve as markers of fibrogenesis; ECM formation exemplified by collagen formation. These markers of collagen turnover (especially collagen types III, IV, and VI) have been studied in different contexts of chronic liver disease and have been found to be particularly useful as biomarkers of fibrotic processes and the degree of fibrosis in different aetiologies.^5^

Liver cirrhosis is the end stage of chronic liver disease and the development of cirrhosis-related complications (*i.e.* ascites, hepatic encephalopathy, gastrointestinal bleeding, and bacterial infections) marks the stage of decompensated liver disease.^6^ One study showed that collagen type III deposition is boosted in patients with acute decompensation (AD) of cirrhosis.^7^ Acute-on-chronic liver failure (ACLF) is a devastating syndrome that occurs in patients with AD, usually triggered by a precipitating event, which leads to the development of (multi-)organ failures. This is associated with a high risk of mortality within 28 days that ranges between 20 and 70%, depending on the number of organ failures.^8^ A central phenomenon in the pathophysiology of ACLF is the systemic inflammatory response, the severity of which negatively impacts outcome,[8], [9], [10] but the underlying mechanisms are unclear. The important role of damage- and pathogen-associated molecular patterns (DAMPs and PAMPs) in the pathogenesis of systemic inflammation in ACLF is under intense investigation, but the role of ECM turnover is unknown.

A wide range of collagen fragments have been identified as bioactive molecules with signalling properties, also referred to as ‘matrikines’. Like other DAMPs, they can signal through the toll-like receptors^4^^,^^11^ and regulate numerous (patho)physiological pathways such as modulating immune response and hepatic regeneration.^11^ Therefore, we aimed to determine the association of biomarkers of ECM turnover with the presence of ACLF. In addition, we aimed to study whether these markers are associated with the severity of systemic inflammation, cell death, organ failures, and mortality.

## Patients and methods

### Study cohorts

**Fig. 1:**
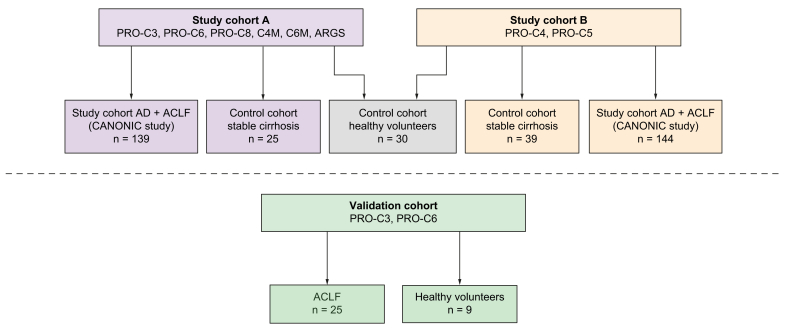
Summary of the different patient cohorts in which markers of ECM turnover were assessed. ACLF, acute-on-chronic liver failure; AD, acute decompensation; ECM, extracellular matrix.

Plasma ECM turnover markers were studied in 7 cohorts of patients or healthy participants (summarised in Fig. 1).

#### Cohort of patients with AD with or without ACLF

A part of the present study is an ancillary study of the CANONIC study, a European multicentre observational study of patients admitted to hospital with AD.^8^ Written informed consent was obtained from each patient included in this study, and the study protocol conformed to the ethical guidelines of the 1975 Declaration of Helsinki, as evaluated by the local Medical Ethics Committees of the participating centres. In 2011, 1,349 patients hospitalised for AD in 29 European liver units were included in this study. Patients were followed up until 28 days after study enrolment, and demographics, clinical characteristics, and laboratory measurements were collected. Survival data were collected at 28 days and 3, 6, and 12 months. The HCB-IDIBAPS Biobank in Barcelona (Spain) manages the database and storage of biomaterials obtained during this study. We used available plasma samples from a subpopulation of the CANONIC cohort (n = 283) obtained at time of hospital admission (Days 0–2). Because of limited sample volume, 2 distinct cohorts were used for the measurements of the selected ECM turnover biomarkers (*i.e.* study cohorts A and B, Fig. 1). For both cohorts, patients were randomly selected from the CANONIC database. Study cohort A (n = 139) was used for measurement of PRO-C3, PRO-C6, PRO-C8, C4M, C6M, and the neoepitope of aggrecan (ARGS). In addition, we wanted to assess plasma PRO-C4 and PRO-C5 levels. These 2 biomarkers were assessed in study cohort B (n = 144), as a result of insufficient sample amount left in study cohort A (Fig. 1). The results for this cohort are described in the Supplementary information.

The presence of organ failures and ACLF was determined using the European Foundation for the study of Chronic Liver Failure (EF-CLIF) Consortium Organ Failure score (CLIF-C OF score).^12^ In addition to the routine laboratory measurements, we had the availability of several biomarkers of systemic inflammation (cytokines IL-6, IL-8, IL-10, and IL1-RA and macrophage activation marker sCD163), of cell death (nonapoptototic cell death: M65 fragment of keratin-18 [K18] and apoptotic cell death: caspase-cleaved M30 component of K18 [cK18]), and of kidney and liver injury (urinary neutrophil gelatinase-associated lipocalin [NGAL]).

#### Control cohorts

The study involved archived plasma samples from 2 control cohorts: (i) 30 healthy volunteers and (ii) 64 patients with stable cirrhosis (SC; samples from n = 25 patients were used as controls for cohort A, and samples from n = 39 were used as controls for cohort B). The SC cohort consisted of patients under follow-up in the outpatient clinic with a clinical, histological, or radiological diagnosis of liver cirrhosis (Child-Pugh score ≤10).^13^ The study was approved by the joint University College London (UCL)/UCL Hospitals (UCLH) Committees (http://www.isrctn.com/ISRCTN62619436), and written informed consent was obtained from each patient included in the study (Fig. 1).

#### Validation cohort

To validate the findings obtained in study cohort A, we used a cohort consisting of 25 patients admitted with ACLF at the Royal Free Hospital, London, UK, and 9 healthy volunteers. These patients were included in an ancillary study of the ongoing DASIMAR trial (ClinicalTrials.gov Identifier: NCT01071746; the Non-interventional Study [NIS]). Written informed consent was obtained from each included patient, and the study was approved by the Joint UCL/UCLH Committees on the Ethics of Human Research (Committee A), with Research Ethics Committee reference number 08/H0714/8. Clinical characteristics and plasma samples were collected at Days 1, 4, and 7 from hospital admission. Survival data were collected at Days 28 and 90 from hospital admission. The diagnosis of organ failures and ACLF in this cohort was also determined using the CLIF-C OF score.

#### Cohort for hepatic immunohistochemistry of PRO-C6 and alpha-smooth muscle actin

To investigate whether ACLF is associated with increased expression of PRO-C6 in the liver, we performed immunohistochemistry (IHC) of PRO-C6 on paraffin-embedded liver biopsies from alcoholic patients with cirrhosis hospitalised with AD (n = 7) or ACLF (n = 5) and received a diagnostic liver biopsy for routine diagnostic purposes. In addition, we performed an alpha-smooth muscle actin (α-SMA) IHC for these patients to understand whether PRO-C6 expression is co-localised with activated HSCs. The methods of PRO-C6 and α-SMA IHC are described in the Supplementary information. This study was approved by the London–Hampstead Research Ethics Committee (07/Q0501/50) and was in compliance with the Declaration of Helsinki.

### Measurements of ECM turnover markers

In study cohort A, we measured the following 6 ECM turnover markers in EDTA plasma using an ELISA-based technology: markers of collagen type III, VI, and VIII formation (PRO-C3,^14^ PRO-C6,^15^ and PRO-C8^16^), markers of collagen type IV and VI degradation (C6M^17^ and C4M^18^), and degraded aggrecan (ARGS^19^) according to the manufacturer (Nordic Bioscience A/S, Herlev, Denmark). Additionally, we measured markers of type IV (PRO-C4) and V (PRO-C5^20^) collagen formation in study cohort B also using assays manufactured by Nordic Bioscience (Supplementary CTAT Table).

### Statistical analysis

The data are presented as mean ± SD when normally distributed or as median (IQR) when following a skewed distribution. Discrete variables are shown as counts (percentage, %). A *p* value of <0.05 was considered statistically significant. Data analysis was performed using R Statistical Software, version 4.0.4. (R Foundation for Statistical Computing, Vienna, Austria).^21^

For the association analysis, we first assessed homogeneity of variances across groups using Bartlett’s test (3 groups) or the *F* test (2 groups). This was followed by a 1-way ANOVA or Welch’s test (3 groups) or the *t* test with equal or unequal variances (2 groups). In case of categorical variables, Fisher’s exact test was applied.

Spearman’s rank order correlation analysis was applied to identify correlations between ECM turnover markers and liver and kidney function parameters, markers of cell death and inflammation, and prognostic scoring systems.

For the survival analysis in the study cohort, competing-risk regression models according to the method of Fine and Gray^22^ were designed to assess the prognostic value of ECM turnover markers in predicting short-term mortality (at 28 and 90 days). In these models, we adjusted for interdependence by considering liver transplantation as a competing-risk factor for mortality. In the validation cohort, no patients received a liver transplantation within 90 days of hospital admission. Therefore, survival analysis in this cohort (28- and 90-day mortality) was performed using Cox proportional hazards regression analysis. Variables with a *p* value of <0.05 in univariate analysis were included in the multivariate analysis, limited to 1 variable per 10 events (mortality) according to the ‘1 in 10 rule’ to avoid overfitting. As we were especially interested in whether the ECM turnover markers would be of prognostic significance, independently of the established prognostic scoring systems in patients with AD and ACLF (*i.e.* CLIF-C AD and CLIF-C ACLF), these scores were prioritised for use in multivariate models. Owing to log-normal distribution of ECM turnover markers, these variables were log-transformed before survival analysis.

To assess the predictive ability of plasma ECM turnover markers with 28- and 90-day mortality, the Gonen and Heller concordance probability estimates (CPEs) were calculated using Cox proportional hazards models. With the coordinates of the receiver operating characteristic (ROC) curves, we calculated the optimum cut-off points of ECM turnover markers in predicting 28- and 90-day mortality using the Youden index. Subsequently, Kaplan–Meier curves were plotted, and transplant-free survival between patients with plasma levels below and above these cut-off points was compared using the log-rank test.

## Results

### Patient characteristics

Baseline demographics and clinical characteristics of the patients with AD and ACLF in the CANONIC study cohort A are shown in Table 1. Of the 139 patients, 35 (25.2%) had ACLF at the time of hospital admission; 19 (54.3%) with ACLF grade I, 12 (34.3%) with ACLF grade II, and 4 (11.4%) with ACLF grade III. Patients with ACLF had significantly more severe liver and kidney injury (bilirubin, international normalised ratio [INR], creatinine, and NGAL), systemic inflammation (IL-8, IL-10, and sCD163), and higher circulating levels of cell death markers K18 and cK18, compared with those without ACLF. Prognostic scores, such as the model for end-stage liver disease (MELD) and the CLIF-C OF score, were also significantly higher in patients with ACLF than in patients with AD without ACLF. A comparison between the baseline characteristics of the cohort of patients with SC, AD, and ACLF is shown in Table S1. As expected, patients with AD with or without ACLF had significantly more severe liver disease compared with those with SC, whereas there were no significant differences in age, sex, and aetiology. The baseline characteristics for study cohort B are presented in Table S2.

**Table 1:** **Baseline characteristics of study cohort A**.

Variables	All patients (n = 139)	AD (n = 104)	ACLF (n = 35)	*p* value∗
Age	57.0 ± 12.2	57.8 ± 12.7	54.9 ± 10.8	0.244
Male sex, n (%)	90 (64.7)	67 (64.4)	23 (65.7)	1
Aetiology of cirrhosis†, n (%)				0.328
Alcohol	48 (41.7)	33 (38.4)	15 (51.7)	
Viral	41 (35.7)	34 (39.5)	7 (24.1)	
Alcohol + viral	11 (9.6)	7 (8.1)	4 (13.8)	
Other	15 (13.0)	12 (14.0)	3 (10.3)	
Clinical features, n (%)				
Ascites	80.0 (86)	49.0 (83.1)	31.0 (91.2)	0.361
Bacterial infection	32.0 (24.8)	21.0 (22.1)	11.0 (32.4)	0.253
ACLF grade I	19 (13.7)	n.a.	19 (54.3)	n.a.
ACLF grade II	12 (8.6)	n.a.	12 (34.3)	n.a.
ACLF grade III	4 (2.9)	n.a.	4 (11.4)	n.a.
Organ failures, n (%)				
Liver	26 (18.7)	7 (6.7)	19 (54.3)	**<0.001**
Renal	18 (12.9)	0 (0)	18 (51.4)	**<0.001**
Cerebral	4 (2.9)	0 (0)	4 (11.4)	**0.004**
Circulatory	6 (4.3)	0 (0)	6 (4.3)	**<0.001**
Respiratory	1 (0.7)	0 (0)	1 (2.9)	0.252
Coagulation	15 (10.8)	6 (5.8)	9 (25.7)	**0.003**
Routine biochemistry				
WCC (×10^9^/L)	6.0 (4.1–8.7)	5.7 (4.1–8.3)	6.6 (4.6–10.2)	0.109
Bilirubin (μmol/L)	82.1 (41.0–153.0)	61.6 (37.0–119.6)	207.8 (46.6–365.1)	**<0.001**
Albumin (g/dl)	2.8 ± 0.5	2.8 ± 0.5	2.8 ± 0.5	0.950
INR	1.6 (1.3–2.1)	1.5 (1.3-1.8)	2.1 (1.5–2.5)	**<0.001**
Creatinine (μmol/L)	84.0 (61.9–123.8)	74.3 (60.1–103.9)	185.2 (100.8–257.9)	**<0.001**
Prognostic and organ failure scores				
MELD	19.5 (15.0–25.0)	17.0 (14.0-21.0)	29.5 (25.0–32.5)	**<0.001**
CLIF-C OF	7.0 (6.0–9.0)	7.0 (6.0–8.0)	10.0 (9.0–11.0)	**<0.001**
CLIF-C AD	51.9 ± 9.5	51.9 ± 9.5	n.a.	n.a.
CLIF-C ACLF	46.5 (42–50)	n.a.	46.5 (42–50)	n.a.
Biomarkers of inflammation, cell death				
IL-6 (pg/ml)	22.6 (9.7–43.4)	22.6 (9.2–37.1)	22.5 (11.8–62.7)	0.166
IL-8 (pg/ml)	45.6 (23.7–110.0)	38.2 (21.2–69.2)	105.7 (28.4–220.0)	**0.002**
IL-10 (pg/ml)	3.0 (1.5–10.9)	2.5 (1.2–8.8)	7.2 (2.0–13.0)	**0.017**
IL-1RA (pg/ml)	8.5 (5.4–17.1)	8.3 (5.3–15.4)	10.3 (6.4–30.7)	0.062
sCD163 (mg/ml)	8.9 (5.5–13.9)	7.6 (4.6–11.2)	14.0 (10.3–19.4)	**<0.001**
NGAL (ng/ml)	28.6 (12.1–99.2)	19.4 (10.3–48.4)	110.9 (35.1–257.2)	**<0.001**
cK18 (U/L)	1019.2 (725.1–1,362.7)	845.1 (704.1–1,248.5)	1,477.1 (1,131.1–5,310.6)	**0.020**
K18 (U/L)	687.4 (288.4–2,349.5)	576.7 (230.3–1,222.9)	2,349.5 (846.3–3,755.4)	**0.012**

ACLF, acute-on-chronic liver failure; AD, acute decompensation; cK18, caspase-cleaved keratin 18; CLIF-C ACLF, CLIF Consortium Acute-on-Chronic Liver; CLIF-C AD, CLIF Consortium Acute Decompensation; CLIF-C OF, CLIF Consortium Organ Failure; INR, international normalised ratio; K18, keratin 18; MELD, model for end-stage liver disease; NGAL, neutrophil gelatinase-associated lipocalin; WCC, white cell count.

∗ *p* values represent comparison of AD *vs*. ACLF calculated using the *t* test or Fisher’s exact test when appropriate; values in bold denote statistical significance (*p* <0.05).

† Information on aetiology was missing for 24 patients.

### Study cohort A

#### ECM turnover markers

**Fig. 2:**
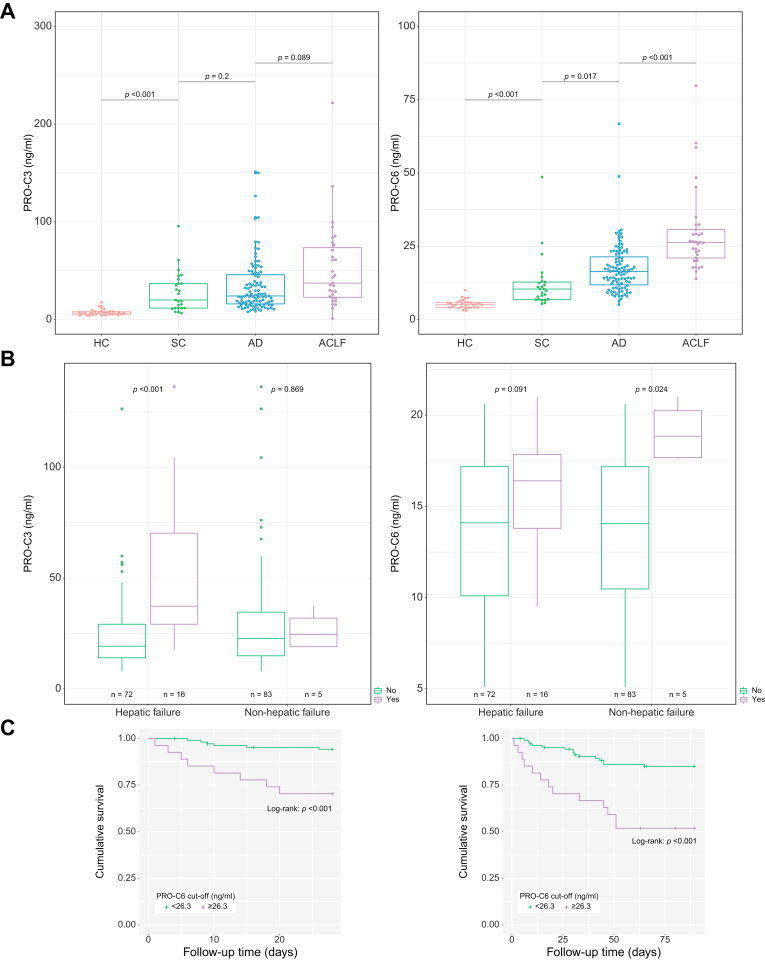
Relationship between plasma concentrations of PRO-C3/PRO-C6 and clinical outcomes. (A) Plasma concentrations of PRO-C3 and PRO-C6 in the study cohort A and control cohorts, (B) comparison of plasma PRO-C3 and PRO-C6 levels in patients with and without hepatic failure and with and without non-hepatic failure, and (C) Kaplan–Meier curves of 28- and 90-day transplant-free survival stratified according to high *vs*. low plasma PRO-C6 levels. T test was used to test for differences between groups, log-rank test was used to compare survival distributions. ACLF, acute-on-chronic liver failure; AD, acute decompensation; HC, healthy control; SC, stable cirrhosis.

In Table 2, plasma concentrations of the panel of ECM turnover markers in study cohort A and control cohorts are presented. For each of the markers, significantly higher plasma levels were found in the cohort of patients with AD/ACLF than in healthy controls. For PRO-C3 and PRO-C6, a stepwise increase was observed between patients with SC, AD, and ACLF (Fig. 2A and Table 2). In contrast, markers of collagen degradation (C4M and C6M) were similar between patients with AD and ACLF. As we found an association of circulating PRO-C3 and PRO-C6 with the presence of ACLF, further analyses were focused on these 2 markers.

**Table 2:** **Plasma levels of ECM turnover markers in the study cohort A and control cohorts**.

Cohort	PRO-C3 (ng/ml)	PRO-C6 (ng/ml)	PRO-C8 (ng/ml)	ARGS (pM)	C6M (ng/ml)	C4M (ng/ml)
Healthy controls (n = 30)	6.7 (5.2–8.4)	5.1 (4.1-5.9)	1.5 (1.2–1.7)	278.3 (234.2–311.9)	6.1 (6.0–8.6)	18.9 (16.2–20.9)
Stable cirrhosis (n = 25)	20.0 (11.7–36.8)	10.4 (6.9-12.8)	n.a.	n.a.	n.a.	n.a.
AD + ACLF (n = 139)	26.2 (17.7–49.4)	17.8 (13.7–24.6)	2.9 (2.1–4.4)	458.2 (302–683.1)	17.0 (13.2–23.4)	25.1 (18.8–31.0)
***p* value**	**<0.001**∗**/<0.001**†	**<0.001/ <0.001**†	**<0.001**†	**0.002**†	**<0.001**†	**<0.001**†
AD (n = 104)	24.1 (16.0–45.8)	16.4 (11.8–21.4)	3.0 (2.1–4.6)	458.0 (292.0–656.3)	17.0 (13.2–23.7)	25.6 (19.4–31.7)
ACLF (n = 35)	37.3 (22.8–73.6)	26.3 (21.3–30.7)	2.8 (2.1–3.6)	487.7 (349.2–751.0)	17.0 (12.4–23.0)	24.0 (17.3–29.8)
***p* value**	0.089	**<0.001**	0.082	0.619	0.507	0.461
ACLF grade I (n = 19)	29.4 (22.1–53.0)	24.2 (20.1–28.9)	2.8 (2.6–3.6)	437.0 (337.6–669.3)	17.5 (13.4–23.5)	22.7 (18.3–32.0)
ACLF grade II (n = 12)	53.1 (26.3–76.9)	26.6 (23.3–29.7)	2.7 (2.2–4.2)	550.0 (407.2–758.4)	17.1 (12.2–22.9)	26.4 (17.1–28.5)
ACLF grade III (n = 4)	78.7 (58.0–82.1)	53.5 (40.7–59.1)	2.0 (1.8–2.9)	914.2 (384.5–1888.4)	14.2 (11.8–19.2)	20.4 (12.4–33.0)
***p* value**‡	0.615	0.335	0.993	0.158	0.800	0.721

Values in bold denote statistical significance (*p* <0.05).

ACLF, acute-on-chronic liver failure; AD, acute decompensation; ECM, extracellular matrix.

∗ One-way ANOVA or Welch’s test over the 3 groups.

† Two-sample *t* test between healthy controls and AD + ACLF.

‡ Two-sample *t* test between ACLF grade I and grades II + III.

Firstly, the association of PRO-C3 and PRO-C6 with hepatic (*i.e.* liver and coagulation) failure *vs.* non-hepatic (*i.e.* kidney, brain, circulatory, and respiratory) failure, as defined by the CLIF-C OF score, was investigated. Both PRO-C3 and PRO-C6 were higher in patients with hepatic failure than in those without (*p* <0.001 and *p* = 0.091, respectively, Fig. 2B). For PRO-C6, but not for PRO-C3, patients with non-hepatic failure had significantly higher plasma levels compared with those without (*p* = 0.024, Fig. 2B).

#### Correlation of PRO-C3 and PRO-C6 with organ injury, inflammation, and cell death

In Table 3, the results of the Spearman rank order correlation analysis are presented. PRO-C3 correlated well with markers of liver function (bilirubin and INR), inflammation (IL-8, IL1-RA, and sCD163), and cell death (K18 and cK18) and with the MELD and CLIF-C OF score. Similarly, PRO-C6 correlated significantly with markers of liver function (bilirubin and INR) and inflammation (IL-8, IL-10, sCD163) and MELD and CLIF-C OF scores. However, unlike PRO-C3, PRO-C6 also correlated with markers of kidney injury (creatinine and NGAL) and the CLIF-C AD score. In contrast to PRO-C3, PRO-C6 did not correlate with plasma levels of the cell death markers K18 and cK18. Correlations of the other assessed ECM turnover markers with liver function, inflammation, cell death, and prognostic scores are described in Table S3.

**Table 3:** **Spearman rank-order correlation analysis for PRO-C3 and PRO-C6 with markers of organ injury, inflammation, cell death, and prognosis in the study cohort A**.

Variables	PRO-C3		PRO-C6	
	Spearman *r*	*p* value	Spearman *r*	*p* value
Liver and kidney injury				
Bilirubin	0.395	**<0.001**	0.266	**0.003**
Albumin	−0.104	0.284	−0.035	0.717
INR	0.302	**0.001**	0.270	**0.002**
Creatinine	−0.057	0.536	0.529	**<0.001**
NGAL	0.137	0.153	0.423	**<0.001**
Inflammation				
WCC	0.141	0.126	0.067	0.462
IL-6	0.057	0.599	0.119	0.264
IL-8	0.429	**<0.001**	0.311	**0.003**
IL-10	0.083	0.445	0.207	0.05
IL-1RA	0.210	0.05	0.185	0.079
sCD163	0.420	**<0.001**	0.477	**<0.001**
Cell death				
cK18	0.326	**0.037**	0.167	0.279
K18	0.349	**0.027**	0.113	0.475
Prognostic scores				
MELD	0.367	**<0.001**	0.514	**<0.001**
CLIF-C AD∗	0.138	**0.197**	0.241	**0.022**
CLIF-C ACLF†	0.336	0.087	0.176	0.360
CLIF-C OF	0.289	**0.002**	0.409	**<0.001**

Values in bold denote statistical significance (*p* <0.05).

ACLF, acute-on-chronic liver failure; AD, acute decompensation; cK18, caspase-cleaved keratin 18; CLIF-C ACLF, CLIF Consortium Acute-on-Chronic Liver; CLIF-C AD, CLIF Consortium Acute Decompensation; CLIF-C OF, CLIF Consortium Organ Failure; INR, international normalised ratio; K18, keratin 18; MELD, model for end-stage liver disease; NGAL, neutrophil gelatinase-associated lipocalin; WCC, white cell count.

∗ This analysis could be performed in patients with AD only (n = 104).

† This analysis could be performed in patients with ACLF only (n = 35).

#### Prognostic value of circulating PRO-C3 and PRO-C6

After 28 days of study inclusion, 15 (10.8%) patients had died and 4 (2.9%) underwent transplantation. At 90 days, 29 (20.9%) patients had died and 11 (7.9%) had received a liver transplant. Results of the univariate competing-risk survival analysis at 28 and 90 days are shown in Table 4. At both time points, PRO-C6 was significantly associated with mortality, whereas PRO-C3 was not. However, in multivariate analysis, PRO-C6 was found not to be an independent prognostic factor in models together with the different prognostic scores (CLIF-C OF and CLIF-C ACLF) at both time points (Table S4).

**Table 4:** **Univariate competing-risk regression analysis for 28- and 90-day mortality in the study cohort A**.

	28-day mortality (15 died, 4 underwent transplantation)		90-day mortality (29 died, 11 underwent transplantation)	
Variable	HR (95% CI)	*p* value	HR (95% CI)	*p* value
Age	1.005 (0.965–1.047)	0.806	1.021 (0.997–1.046)	0.091
Collagen formation markers				
Log(PRO-C3)	1.349 (0.633–2.874)	0.438	1.280 (0.653–2.509)	0.472
Log(PRO-C6)	6.168 (2.366–16.080)	**<0.001**	3.495 (1.509–8.093)	**0.003**
Liver and kidney injury				
Log(Bilirubin)	4.100 (2.145–7.835)	**<0.001**	3.201 (1.912–5.359)	**<0.001**
Albumin	0.337 (0.102–1.107)	0.073	0.308 (0.124–0.767)	**0.011**
Log(INR)	11.692 (2.666–51.272)	**0.001**	12.092 (4.261–34.311)	**<0.001**
Log(Creatinine)	3.596 (1.662–7.78)	**0.001**	2.718 (1.476–5.005)	**0.001**
Log(NGAL)	2.298 (1.628–3.243)	**<0.001**	1.896 (1.403–2.563)	**<0.001**
Inflammation				
Log(WCC)	4.134 (1.385–12.337)	**0.011**	3.699 (1.517–9.019)	**0.004**
Log(IL-6)	1.517 (1.165–1.975)	**0.002**	1.283 (0.964–1.706)	0.087
Log(IL-8)	2.355 (1.527–3.63)	**<0.001**	1.686 (1.205–2.359)	**0.002**
Log(IL-10)	1.310 (0.985–1.744)	0.064	1.308 (1.038–1.648)	**0.023**
Log(IL-1RA)	1.743 (1.267–2.397)	**0.001**	1.480 (1.089–2.013)	**0.012**
Log(sCD163)	7.512 (2.715–20.787)	**<0.001**	6.528 (2.893–14.731)	**<0.001**
Cell death				
Log(cK18)	2.500 (1.431–4.366)	**0.001**	2.240 (1.521–3.299)	**<0.001**
Log(K18)	1.936 (1.169–3.207)	**0.01**	1.826 (1.25–2.669)	**0.002**
Prognostic scores				
MELD	1.180 (1.1–1.266)	**<0.001**	1.175 (1.118–1.235)	**<0.001**
CLIF-C AD∗	1.259 (1.109–1.429)	**<0.001**	1.157 (1.095–1.221)	**<0.001**
CLIF-C ACLF†	1.136 (1.04–1.24)	**0.005**	1.168 (1.085–1.257)	**<0.001**
CLIF-C OF	1.69 (1.385–2.061)	**<0.001**	1.579 (1.325–1.882)	**<0.001**

Values in bold denote statistical significance (*p* <0.05).

ACLF, acute-on-chronic liver failure; AD, acute decompensation; cK18, caspase-cleaved keratin 18; CLIF-C ACLF, CLIF Consortium Acute-on-Chronic Liver; CLIF-C AD, CLIF Consortium Acute Decompensation; CLIF-C OF, CLIF Consortium Organ Failure; HR, hazard ratio; INR, international normalised ratio; K18, keratin 18; MELD, model for end-stage liver disease; NGAL, neutrophil gelatinase-associated lipocalin; WCC, white cell count.

∗ This analysis could be performed in patients with AD only (n = 104), of which 5 died and 2 underwent transplantation at 28 days and 15 died and 5 underwent transplantation at 90 days.

† This analysis could be performed in patients with ACLF only (n = 35), of which 10 died and 2 underwent transplantation at 28 days and 14 died and 6 underwent transplantation at 90 days.

In correspondence with the findings in the univariate analysis, CPE for PRO-C3 (around 0.55 at both 28 and 90 days) pointed towards a poor predictive ability of this biomarker in the study cohorts of patients with AD and ACLF (Table S5). CPEs for PRO-C6 were 0.704 (95% CI 0.625–0.783) and 0.653 (95% CI 0.578–0.728) at 28 and 90 days, respectively. The CPEs of the CLIF-C OF score were slightly improved with 0.028 and 0.022 points by incorporating log(PRO-C6) in this score (28 days: 0.698–0.726; 90 days: 0.680–0.702, Table S5). The optimal cut-off point of 26.3 ng/ml for plasma PRO-C6 in predicting 28- and 90-day mortality was calculated using the Youden index. Using this cut-off point, we plotted Kaplan–Meier curves and compared them using the log-rank test. Patients with a baseline PRO-C6 level below this cut-off point had a significantly better transplant-free survival at both time points (Fig. 2C).

### IHC of PRO-C6 and α-SMA in AD with and without ACLF

**Fig. 3:**
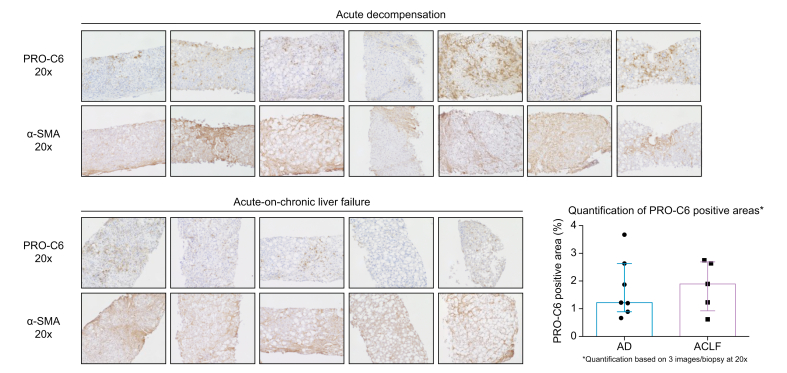
Immunohistochemistry of PRO-C6 and α-SMA in liver biopsies of patients with alcoholic hepatitis with AD (n = 5) and ACLF (n = 7). Magnification 20×. α-SMA, alpha-smooth muscle actin; ACLF, acute-on-chronic liver failure; AD, acute decompensation.

We observed high expression of PRO-C6 in liver biopsies of patients with AD and alcoholic cirrhosis who were admitted with or without ACLF. The patients with ACLF seemed to have more intense PRO-C6 expression as compared with those with AD, although not statistically significant. In consecutive sections, the α-SMA expression seemed to be co-localised with PRO-C6, although α-SMA was more abundantly present (Fig. 3 and Fig. S1). These data suggest that PRO-C6 is primarily present around activated HSCs and therefore in areas with an ongoing wound healing response. However, other factors seem to play a role, as areas with high PRO-C6 but low α-SMA expression were also observed. Methods of PRO-C6 and α-SMA IHC are described in the Supplementary information.

### Validation cohort

The baseline characteristics of the patients included in the validation cohort are shown in Table S6. All patients included in the validation cohort had ACLF at time of hospital admission. Nine (36%) had ACLF grade I, 8 (32%) grade II, and 8 (32%) grade III. When comparing the subpopulation with ACLF of study cohort A with the validation cohort, we found that the latter had a higher proportion of patients with ACLF grade III (32% *vs*. 11.4%), which was also reflected by significantly higher CLIF-C OF scores (11 [10–13] *vs*. 10 [9–11], *p* <0.001), creatinine (*p* = 0.033), and white cell count (WCC) (*p* = 0.002). Nevertheless, liver function and presence of individual organ failures were largely similar between the cohorts.

**Fig. 4:**
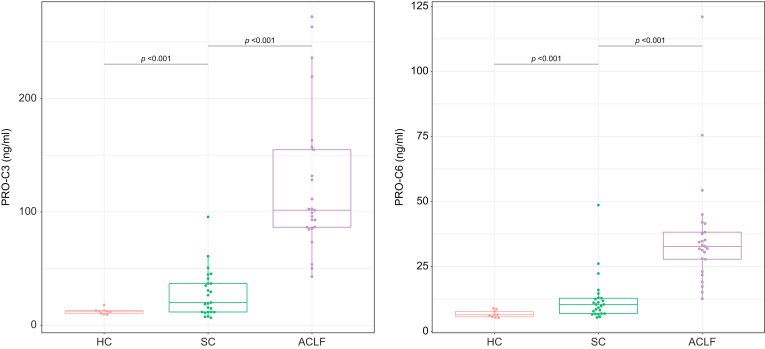
Plasma concentrations of PRO-C3 and PRO-C6 in the validation cohort of patients with ACLF and validation control cohorts. T test was used to compare each pair of groups. ACLF, acute-on-chronic liver failure; HC, healthy control; SC, stable cirrhosis.

Based on the findings in the study cohort, we only measured PRO-C3 and PRO-C6 in the validation cohort. These measurements validated the findings in the study cohort A that patients with ACLF had significantly higher levels of PRO-C3 and PRO-C6 as compared with patients with SC and healthy volunteers (Fig. 4A). After 28 days of hospital admission, 12/25 (48%) of patients in the validation cohort had died. This increased to 16 (64%) after 90 days. None of the patients received a liver transplant within 90 days. The mortality rates at 28 and 90 days were not significantly different between the study and validation cohorts (*p* = 0.175 and *p* = 0.115, respectively, Table S6).

Similar to the findings in the survival analysis of the study cohort, PRO-C6 was associated with 28-day and 90-day mortality in the univariate Cox-regression analysis, whereas PRO-C3 was not (Table 5). PRO-C6 levels measured at Days 4 and 7 were more strongly associated with mortality at both 28 and 90 days than when measured at baseline. In multivariate analysis, plasma PRO-C6 measured at Day 4 was a predictor of 90-day mortality, independently of the CLIF-C OF score (Table S7).

**Table 5:** **Cox proportional hazards regression analysis in the validation cohort**.

	28-day mortality (12 died, 0 underwent transplantation)		90-day mortality (16 died, 0 underwent transplantation)	
Variable	HR (95% CI)	*p* value	HR (95% CI)	*p* value
Age	1.045 (0.98–1.113)	0.178	1.056 (0.997–1.119)	0.065
ECM turnover markers				
Log(PRO-C3_day1)	0.894 (0.277–2.888)	0.852	0.683 (0.244–1.915)	0.469
Log(PRO-C3 day4)	2.103 (0.311–14.215)	0.446	2.893 (0.645–12.982)	0.165
Log(PRO-C3_day7)	1.976 (0.355–10.997)	0.437	2.167 (0.495–9.496)	0.305
Log(PRO-C6 day1)	4.396 (1.147–16.851)	**0.031**	4.069 (1.262–13.122)	**0.019**
Log(PRO-C6_day4)	26.613 (2.737–258.751)	**0.005**	31.327 (4.408–222.645)	**0.001**
Log(PRO-C6 day7)	7.216 (1.721–30.249)	**0.007**	9.216 (2.549–33.324)	**0.001**
Liver and kidney function				
Bilirubin	1.000 (0.997–1.003)	0.829	0.999 (0.997–1.002)	0.465
Albumin	1.049 (0.935–1.176)	0.417	1.125 (1.023–1.236)	**0.015**
INR	2.332 (0.655–8.299)	0.191	2.279 (0.754–6.886)	0.144
Log(Creatinine)	2.732 (0.941–7.93)	0.065	3.035 (1.192–7.73)	**0.020**
Inflammation				
Log(WBC)	1.065 (0.379–2.994)	0.906	1.134 (0.473–2.718)	0.778
Prognostic scores				
CLIF-C OF Day 1	1.449 (1.128–1.861)	**0.004**	1.504 (1.208–1.872)	**<0.001**
CLIF-C OF Day 4	1.418 (1.121–1.793)	**0.004**	1.336 (1.100–1.623)	**0.004**
CLIF-C OF Day 7	1.710 (1.275–2.295)	**<0.001**	1.795 (1.333-2.417)	**<0.001**
CLIF-C ACLF Day 1	1.105 (1.030–1.185)	**0.005**	1.126 (1.056–1.201)	**<0.001**
CLIF-C ACLF Day 4	1.090 (1.027–1.157)	**0.005**	1.081 (1.029–1.137)	**0.003**
CLIF-C ACLF Day 7	1.114 (1.046–1.186)	**0.001**	1.117 (1.051–1.188)	**<0.001**

Values in bold denote statistical significance (*p* <0.05).

CLIF-C ACLF, CLIF Consortium Acute-on-Chronic Liver; CLIF-C OF, CLIF Consortium Organ Failure; ECM, extracellular matrix; HR, hazard ratio; INR, international normalised ratio; WBC, white blood cell.

The poor predictive ability of PRO-C3 for both 28- and 90-day mortality observed in the study cohort was confirmed in the validation cohort (28 days: CPE 0.515, 95% CI 0.354–0.677; 90 days: CPE 0.552, 95% CI 0.413–0.690). CPE for PRO-C6 for mortality in the validation cohort was 0.667 (95% CI 0.538–0.796) at 28 days and 0.660 (95% CI 0.542–0.777) at 90 days (Table S8). PRO-C6 measured at Day 4 or 7 had an improved predictive ability at both time points, as compared with that at baseline (Day 4: CPE 0.773 and 0.781, respectively; Day 7: CPE 0.751 and 0.770, respectively; Table S8). This agrees with a previous study that found disease course and outcome in patients admitted with ACLF to be most accurately assessed by the disease stage at Days 3–7, as compared with the clinical condition at baseline.^23^ In addition, the change in PRO-C6 between Days 1 and 4 and between Days 1 and 7 had a better predictive ability than had a single baseline PRO-C6 measurement (Table S8). In Kaplan–Meier analysis, high PRO-C6 at Day 4 of hospital admission and high delta PRO-C6 between Days 1 and 4 of hospital admission showed significant worse transplant-free survival (*p* <0.001 and *p* = 0.002, Fig. S2).

## Discussion

This study describes for the first time that ACLF is associated with marked increases in plasma levels of biomarkers of interstitial ECM formation (PRO-C3 and PRO-C6) but unchanged levels of markers of ECM breakdown (C4M and C6M). This is likely to be clinically relevant, as exacerbated hepatic collagen deposition may result in further deterioration of reserve liver function. Indeed, both plasma PRO-C3 and PRO-C6 were increased in patients with hepatic failure and correlated well with markers of liver function. This is not surprising as both PRO-C3 and PRO-C6 are biomarkers of collagen formation derived from fibroblasts and thus correlate directly to fibroblast activity.^24^ Both markers are well known to be associated with increased severity of fibrosis and portal hypertension.^5^^,^^7^^,^^17^ Interestingly, the plasma levels of the collagen type IV formation marker PRO-C4 were similar between patients with AD and ACLF. Collagen type IV is the main collagen component of the basement membrane, whereas collagen types III and VI are found in the interstitial space. Therefore, it seems that interstitial fibroblast activity is highly related to ACLF, which is a potentially important observation.

Despite the fact that PRO-C3 and PRO-C6 both reflect fibroblast activity, our results suggest distinct roles for PRO-C3 and PRO-C6 in the setting of AD of cirrhosis. Firstly, in contrast to PRO-C3, plasma PRO-C6 was also associated with the presence of extrahepatic organ failures and correlated with markers of kidney function and injury (creatinine and NGAL). Secondly, PRO-C6 was found to be associated with mortality at 28 and 90 days in the univariate competing-risk analysis. High PRO-C6 levels (greater than the optimum cut-off point) were associated with worse transplant-free survival at 28 and 90 days. In contrast, the prognostic and predictive ability of PRO-C3 in this population was much poorer. These discrepancies may be explained by important differences in properties of these 2 collagen formation markers.

PRO-C3 is a biomarker of collagen type III, which is encoded by the COL3A1 gene and belongs to the fibrillar collagen superfamily.^4^^,^^25^ PRO-C3 is an epitope of the N-terminal pro-peptide generated by N-protease ADAMTS-2 and is released during type III collagen maturation.^14^ A wide range of studies have shown that circulating PRO-C3 levels correlate with the severity of fibrosis[26], [27], [28], [29], [30], [31] but not with survival, which was also not found in the present study. The non-fibrillar collagen, type VI collagen, is found near the basement membrane of the ECM and consists of 6 different chains (α1–α6) encoded by 6 different genes (COL6A1–6).^32^ Type VI collagen binds to a wide range of cell types such as fibroblasts, haematopoietic cells, ECM proteins, and collagen fibrils, thereby playing an important role in regulating matrix assembly and organisation.^33^ Cleavage at a still unknown site of the C-terminal pro-peptide of the α3 chain releases the type VI collagen formation marker PRO-C6.

The most important difference between PRO-C3 and PRO-C6 is that PRO-C6 is known to be a bioactive molecule. Some ECM-derived fragments have bioactive signalling properties and regulate numerous biological processes including angiogenesis, migration, proliferation, apoptosis, metastasis, and tumour growth. These signalling fragments are also referred to as ‘matrikines’.^4^ PRO-C6 is such a matrikine and is in that context also called ‘endotrophin’. Early studies of this relatively newly discovered fragment revealed that adipocyte-derived PRO-C6 induces upregulation of pro-fibrotic and pro-inflammatory genes, thereby promoting tissue inflammation, fibrogenesis, insulin resistance, and angiogenesis.[34], [35], [36] In addition to adipose tissue, PRO-C6 has been found to be present in fibrotic kidneys and lungs, and it has been proposed as a promising prognostic marker of outcome of chronic kidney disease[37], [38], [39] and chronic obstructive airway disease.^40^^,^^41^ In the present study, we found high expression of PRO-C6 in liver biopsies of patients with ACLF. The cellular origin of PRO-C6 and whether it is also expressed in the extrahepatic organs in ACLF remains to be investigated. In previous preclinical studies, PRO-C6 has been shown to promote fibrogenesis and to induce JNK-dependent hepatocyte apoptosis and hepatic inflammation on the background of chronic liver injury.^42^^,^^43^ The known differences in tissue distribution and biologic characteristics of PRO-C3 and PRO-C6 may explain the correlation of PRO-C6 levels with extrahepatic organ failure and mortality in patients with ACLF.

*In vivo* studies showed that laminin, elastin, hyaluronan, and collagen-derived fragments can promote processes such as monocyte/macrophage recruitment, neutrophil migration, and cytokine release through toll-like receptor (TLR)2 and TLR4 signalling pathways.^44^ Our study was not designed to address whether circulating PRO-C6 is causally related to the systemic inflammatory response seen in ACLF, and this therefore needs further investigation. Nevertheless, we observed clear correlations between PRO-C3/PRO-C6 and IL-8, IL10, and IL-1RA and the macrophage activation marker sCD163. This may suggest a link between liver inflammation, macrophage activation, and biomarkers of interstitial ECM formation. In line with this, stimulation of fibroblasts with cytokines such as IL-6, likely produced by activated macrophages, has been shown to lead to the production of PRO-C6.^24^ This allows one to hypothesise that this cell type, along with macrophages, may be important in ACLF.^45^^,^^46^

The present study has some important strengths and limitations. This study comprises a very well-defined study cohort of patients with AD and ACLF, derived from the prospectively performed multicentre observational CANONIC trial. In addition, we had the availability of a validation cohort of patients with ACLF, which showed consistent results. An important limitation is the retrospective design of this ancillary study and the absence of serial measurements of PRO-C3 and PRO-C6 in the study cohort. Secondly, the number of patients with ACLF in the study cohort is low, which impairs the statistical assessment of this subgroup, especially the association of PRO-C3 and PRO-C6 with the severity of ACLF. Finally, whether PRO-C6 is a biomarker or has pathophysiological significance in the development of ACLF has not been addressed in the present study and needs to be investigated in future studies.

Collectively, our findings in both the study and validation cohorts consistently show that the collagen formation markers plasma PRO-C3 and PRO-C6 increase with the different stages of liver disease, with a marked upregulation in ACLF as compared with that in AD. The observed increase of ECM formation in ACLF and known properties of PRO-C6 may contribute to its pathogenesis. Furthermore, the association of PRO-C6 with extrahepatic organ failures, inflammation, and mortality in ACLF points toward a role for this relatively newly discovered bioactive molecule in the pathogenesis of ACLF. As both PRO-C3 and PRO-C6 are formation peptides of collagens that are associated with fibroblast activity, this cell type may be important in ACLF. Further research is needed to understand the molecular mechanisms and its suitability as a prognostic marker and/or a therapeutic target.

## Financial support

The biomarker measurements presented in this study were financially supported by the Danish Research Foundation and by 10.13039/100016469Takeda Pharmaceuticals International Co. Signe Holm Nielsen and Ida Lønsmann received funding from 10.13039/100012774Innovation Fund Denmark.

## Authors’ contributions

Study concept and design: SG, WT, MAK, RJ, AJCK. Acquisition of data: AJCK, EA, JM, RM, FA, SM. Analysis and interpretation of data: AJCK, SG, DJL, SHN, FA, MJN, WT, MAK, RJ. Statistical analysis: ID, AJCK, DY, ÀA. Experimental support: IL, AH, DJL, SHN, MJN. Drafting of the manuscript: AJCK. Critical revision of the manuscript for important intellectual content: SG, EA, ID, ILC, AH, DJL, SHN, FA, HG, ÀA, MJN, JM, RM, SM, RM, VA, PA, WT, MAK, RJ. Obtained funding: SG, WT. Study supervision: RJ.

## Data availability statement

The data generated and analysed during the current study are included in this published article or available from the corresponding author on reasonable request.

## Conflicts of interest

AJCK has none to declare. SG has none to declare. EA has none to declare. ID is a full-time employee at Takeda Pharmaceuticals International Co. IL is a full-time employee at Nordic Bioscience. AH has none to declare. SHN is a full-time employee at Nordic Bioscience. MJN is a full-time employee at Nordic Bioscience. HG received an investigator-initiated research grant from Intercept and research grants from AbbVie, ADS AIPHIA Development Services AG (Switzerland), ARLA Food for Health, and the NOVO Nordisk Foundation. HG is on an advisory board at Ipsen. ÀA has none to declare. DY is a full-time employee at Takeda Pharmaceuticals International Co. JM is a co-founder of Yaqrit Ltd. RPM has research collaboration with Yaqrit Ltd., of which he is a co-founder. He also has research collaborations with UCL spin-out Hepyx Ltd. and with Cyberliver Limited. He also has provided a consultancy role to Inventiva Pharmaceuticals. SM has none to declare. FA has none to declare. RM has none to declare. VA is a member of the Yaqrit Scientific Advisory Board. In 2016–2020, PA was on the Biovie Advisory Board and filed a patent application; in 2014–2020, he was invited as a speaker by and received a travel grant from CSL Behring; and in 2018–2019, he was on the Advisory Board at Ferring. DJL is a full-time employee and stockholder at Nordic Bioscience. WT is a full-time employee at Takeda Pharmaceuticals International Co. MAK is a full-time employee and stockholder at Nordic Bioscience. RJ has research collaborations with Yaqrit and Takeda. RJ is the inventor of OPA, which has been patented by the University College London and licensed to Mallinckrodt Pharma. He is also a founder of Yaqrit Ltd., a spinout company from the University College London. He has also co-founded Hepyx Ltd. and Cyberliver Ltd.

Please refer to the accompanying ICMJE disclosure forms for further details.
