# Supplementary material for: Biomarkers of extracellular matrix formation are associated with acute-on-chronic liver failure
Source: JHEP Rep. 2021 Aug 27;3(6):100355. doi: 10.1016/j.jhepr.2021.100355 (PMC8581571; doi:10.1016/j.jhepr.2021.100355)
Supplement: Multimedia component 1 [file mmc1.pdf]

Biomarkers of extracellular matrix formation are associated with acute-on-chronic liver failure

Annarein J.C. Kerbert, Saurabh Gupta, Eman Alabsawy, Iwona Dobler, Ida Lønsmann, Andrew Hall, Signe Holm Nielsen, Mette J. Nielsen, Henning Gronbaek, Àlex Amoros, Dave Yeung, Jane Macnaughtan, Rajeshwar P. Mookerjee, Stewart Macdonald, Fausto Andreola, Richard Moreau, Vicente Arroyo, Paolo Angeli, Diana J. Leeming, William Treem, Morten A. Karsdal, Rajiv Jalan

Table of contents

Supplementary methods....................2

Supplementary figures....................4

Supplementary tables....................7

Supplementary reference....................19

SUPPLEMENTARY METHODS

PRO-C6 immunohistochemistry

Liver sections were deparaffinized in toluene prior to wash in 99% ethanol and blockage with 1.05% H_2O_2 in 99% ethanol. Then, slides were rehydrated in 96-70% ethanol and water. To unmask epitopes, slides were incubated overnight in 60°C water bath in citrate buffer pH 6.0. The following day, they were washed in Tris Buffered Saline (TBS; 50 mM Tris, 150 mM NaCl, pH 7.5), blocked with 0.5% casein in TBS and incubated overnight at 4°C with 2.6 µg/ml monoclonal PRO-C6 antibody. Bound antibody was visualized with super sensitive polymer-HRP IHC detection system from BioGenex (Fremont, CA, USA) according to the manufacturer's instructions. The reaction was stopped with water followed by counterstaining with Mayer's hematoxylin for 30 sec and 10 min rinse in water. Finally, the slides were dehydrated in 96-99% ethanol and toluene prior to mounting in toluene-based glue. DAB-positive image quantification was performed by ImageJ software (version 1.53a) and expressed as relative DAB-positive area (%). Image quantification was performed at 20X magnification and was based on an average of 3 images per biopsy.

α-SMA immunohistochemistry

Liver sections were deparaffinized in xylene and rehydrated in 90-70% ethanol and distilled water. Antigen retrieval was performed by incubating the sections in a Tris EDTA buffer pH 9.0 in the microwave at 640W for 15 minutes. Sections were washed with TBS with 0.04% Tween-20 for 5 minutes. Subsequently, peroxidase blocking in a peroxidase blocking solution (Novolink™ Kit – Leica) was performed for 5 minutes. After washing with TBS, sections were then blocked with protein block (Novolink™ Kit – Leica) for 5 minutes. Sections were then incubated for 1 hour at room temperature

in Dako anti alpha-SMA M085101-2 at 1:500 in TBS. After washing in TBS, this was followed by incubation for 25 minutes in post primary (Novolink^{TM} Kit - Leica), washing in TBS Tween 5 minutes and then 25 minutes incubation in polymer solution (Novolink^{TM} Kit - Leica). After washing in TBS + 0.04% Tween-20, the antibody was visualized with 3,3′ di-amino-benzidine (Novolink^{TM} Kit - Leica). The reaction was stopped by washing in TBS + 0.04% Tween-20 for 5 minutes followed by counter staining with Mayer’s hematoxylin (Novolink^{TM} Kit - Leica). Finally, sections were dehydrated in 70-100% ethanol, cleared with xylene and mounted.

SUPPLEMENTARY FIGURES

Legends to supplementary figures.

Fig. S1. Immunohistochemistry of PRO-C6 and α-SMA in liver biopsies of alcoholic hepatitis patients with (n=5) and without (n=7) ACLF. Magnification 2.5X.

Fig. S2. Kaplan Meier curves of 28-day transplant-free survival stratified according to high vs. low plasma PRO-C6 levels at day 4 of hospital admission and delta PRO-C6 (day 1-4 of hospital admission).

Fig. S1.

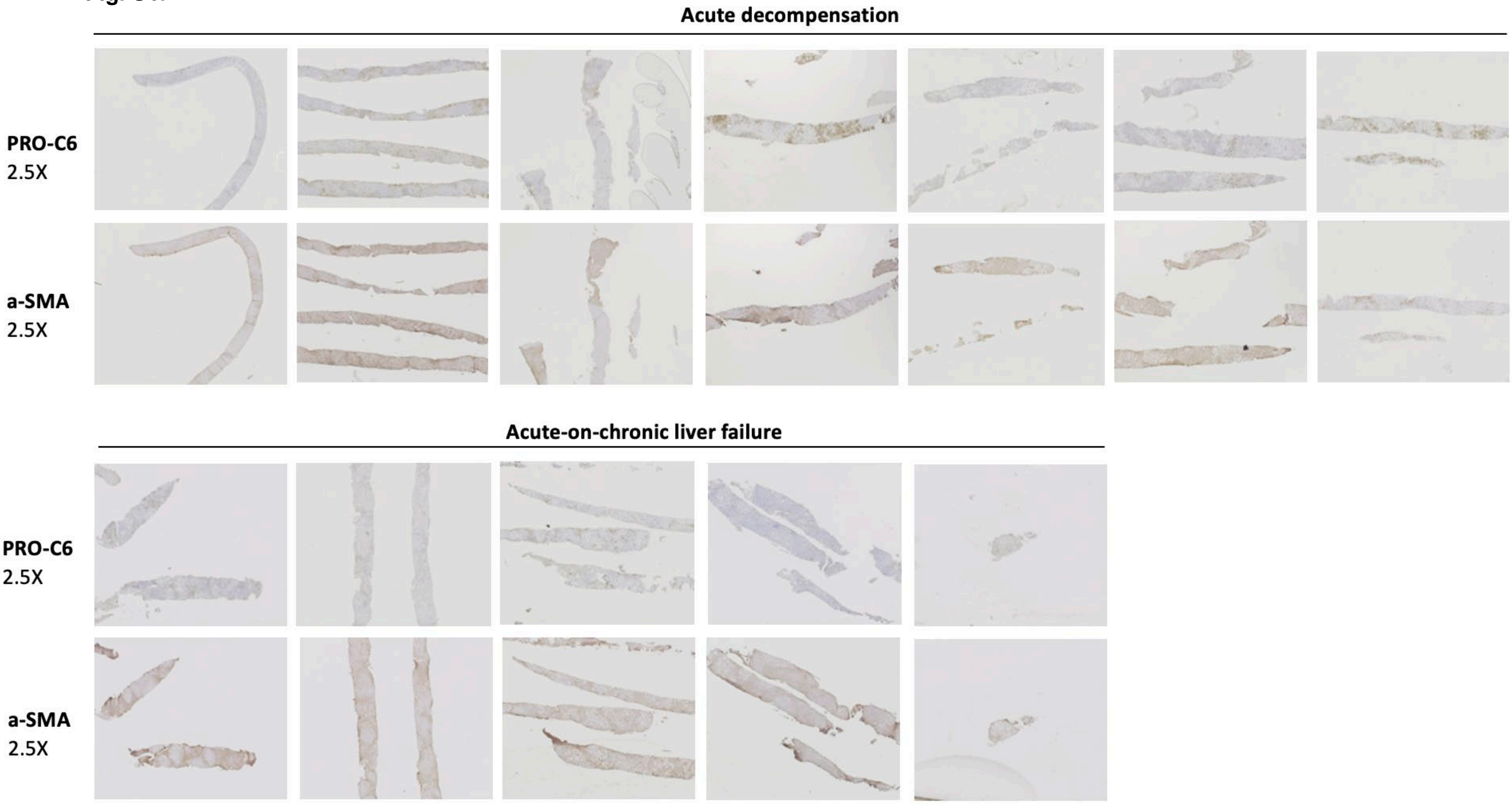
Acute decompensation
PRO-C6
2.5X
a-SMA
2.5X
Acute-on-chronic liver failure
PRO-C6
2.5X
a-SMA
2.5X

Fig. S2.

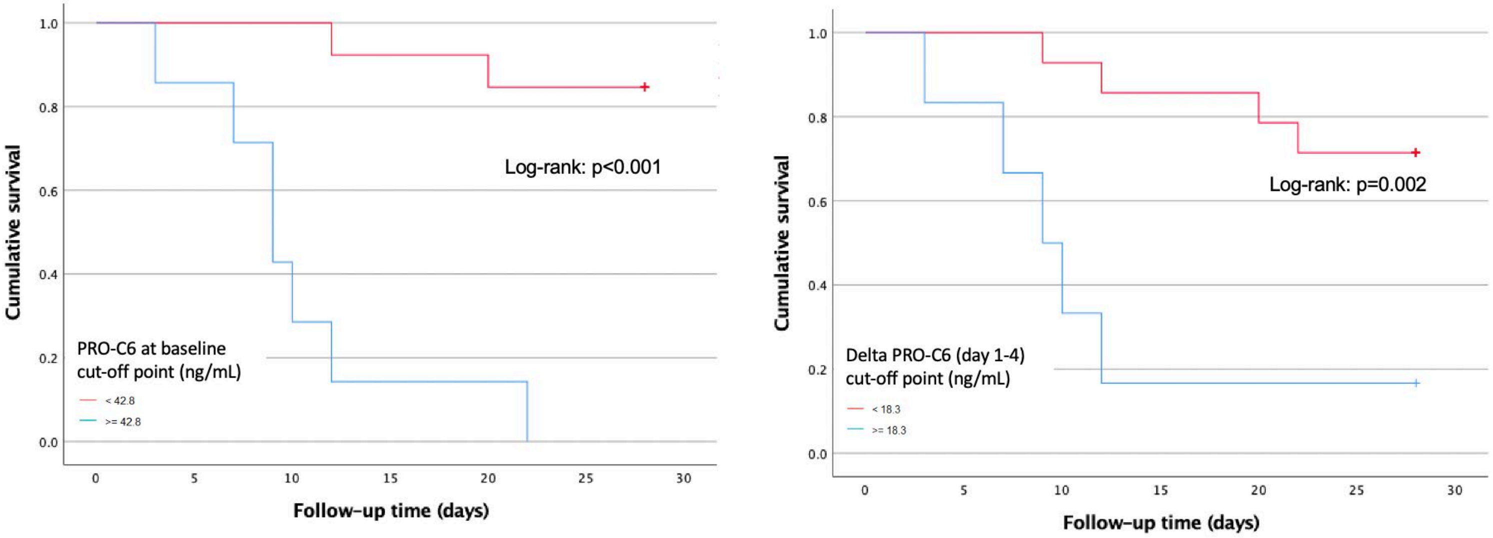
1.0
0.8
0.6
0.4
0.2
0.0
Cumulative survival
Log-rank: p<0.001
PRO-C6 at baseline
cut-off point (ng/mL)
< 42.8
>= 42.8
0
5
10
15
20
25
30
Follow-up time (days)
1.0
0.8
0.6
0.4
0.2
0.0
Cumulative survival
Log-rank: p=0.002
Delta PRO-C6 (day 1-4)
cut-off point (ng/mL)
< 18.3
>= 18.3
0
5
10
15
20
25
30
Follow-up time (days)

SUPPLEMENTARY TABLES

Table S1. Baseline characteristics of the study cohort A compared to the two control cohorts of patients with stable cirrhosis and healthy controls.

Variables	Healthy control (n=30)	Stable cirrhosis (n=25)	AD or ACLF (n=139)	P-value
**Age, mean ± sd**	40.5 ± 12.1	56.5 ± 7.7	57.0 ± 12.2	**<0.001***/ 0.804**
**Male gender, n (%)**	15 (50%)	16 (64%)	90 (64.7%)	0.325^/ 1^^
**Etiology of cirrhosis, n (%)#**				0.637
Alcohol		13 (56.5%)	48 (41.7%)	
Viral		6 (26.1%)	41 (35.7%)	
other		3 (13%)	15 (13%)	
Alcohol + viral		1 (4.3%)	11 (9.6%)	
**Laboratory parameters**				
Bilirubin (umol/L)		15.0 (10.2-20.5)	82.1 (41-153)	**<0.001**
Albumin (g/L)		41.0 (40-43)	29.0 (25-32)	**<0.001**
INR		1.1 (1.0-1.2)	1.6 (1.3-2.1)	**<0.001**
Creatinine (umol/L)		72.0 (65.5-78)	84 (61.9-123.8)	**<0.001**
WCC ($x10^9$/L)		5.8 (4.6-7.3)	6.0 (4.1-8.7)	0.533
MELD score		8.0 (7.0-10.0)	19.5 (15.0-25.0)	**<0.001**

*Welch's test over the 3 groups. **Two sample's t-test 'stable cirrhosis' vs. 'AD or ACLF'.
^Fisher's exact test over 3 groups comparison ^^Fisher's exact test 'stable cirrhosis' vs 'AD or ACLF'.
#Etiology was unknown for 2 (8%) patients in the stable cirrhosis group and for 24 (17.3%) patients in the AD or ACLF group.

Table S2. Baseline characteristics (A) and plasma concentrations of PRO-C4 and PRO-C5 (B) in the study cohort B.

A.

**Variables**	**All patients (n=144)**	**No ACLF (n=116)**	**ACLF (n=28)**	**P-value**
**Age**	58.9 ± 11.3	58.4 ± 11.4	60.7 ± 11.1	0.348
**Male gender**, n (%)	90 (62.5%)	78 (67.2%)	12 (42.9%)	
**Etiology of cirrhosis***, n (%)				0.478
Alcohol	61 (42.4%)	53 (45.7%)	8 (28.6%)	
Viral	27 (18.8%)	25 (21.6%)	2 (7.1%)	
Alcohol + viral	7 (4.9%)	5 (4.3%)	2 (7.1%)	
Other	15 (10.4%)	13 (11.2%)	2 (7.1%)	
**Clinical features**, n (%)				
Ascites	73 (50.7%)	46 (39.7%)	27 (96.4%)	**0.013**
ACLF grade I	16 (57.1%)		16 (57.1%)	n.a.
ACLF grade II	9 (32.1%)		9 (32.1%)	n.a.
ACLF grade III	3 (10.7%)		3 (10.7%)	n.a.
**Organ failures**, n (%)				
Liver	17 (11.8%)	8 (6.9%)	9 (32.1%)	**0.001**
Renal	15 (10.4%)	0	15 (53.6%)	**<0.001**
Cerebral	9 (6.2%)	4 (3.4%)	5 (17.9%)	**0.014**
Circulatory	5 (3.5%)	1 (0.8%)	4 (14.3%)	**0.005**
Respiratory	3 (2.1%)	0	3 (10.7%)	**0.007**
Coagulation	9 (6.2%)	1 (0.8%)	8 (28.6%)	**<0.001**
**Routine biochemistry**				
WCC (x10^9/L)	5.6 (3.8-9.0)	5.5 (3.7-8.4)	7.3 (4.7-9.9)	0.196
Bilirubin (mg/dL)	2.8 (1.6-5.0)	2.7 (1.4-4.4)	4.6 (2.1-12.6)	**0.004**
Albumin (mg/dL)	2.8 (2.6-3.3)	2.8 (2.6-3.2)	2.9 (2.2-3.6)	0.734
INR	1.4 (1.3-1.7)	1.4 (1.3-1.6)	1.6 (1.3-2.6)	**0.006**
Creatinine (mg/dL)	0.9 (0.8-1.4)	0.9 (0.7-1.2)	2.0 (1.1-3.0)	**<0.001**
**Prognostic and organ failure scores**				
MELD	16.0 (13-20.5)	15.0 (12.0-18.2)	25.0 (23-28)	**<0.001**
CLIF-C OF	7.0 (6.0-8.5)	6.0 (6.0-7.0)	10.0 (9.0-11.0)	**<0.001**
CLIF-C AD	50.8 ± 7.7	50.8 ± 7.7		n.a.
CLIF-C ACLF	48.2 ± 8.8		48.2 ± 8.8	n.a.
**Biomarkers of inflammation, cell death**				
IL-6 (pg/mL)	27.5 (15.2-57.5)	26.8 (14.0-47.7)	39.7 (19.5-192.6)	**0.003**
IL-8 (pg/mL)	39.8 (26.0-110.7)	30.5 (21.7-94.9)	73.3 (49.4-157.9)	**<0.001**
IL-10 (pg/mL)	4.7 (2.0-12.4)	3.9 (1.4-9.6)	10.8 (2.7-70.2)	**0.003**
IL1-RA (pg/mL)	14.0 (6.3-29.2)	11.4 (5.0-16.9)	24.2 (12.0-43.0)	**0.002**
sCD163 (mg/mL)	8.8 (5.2-13.6)	7.9 (5.0-12.0)	14.8 (7.4-18.9)	**0.003**
NGAL (ng/mL)	31.9 (12.0-87.1)	26.7 (11.6-59.6)	111.3 (47.4-261.2)	**0.001**
cK18 (U/L)	949.0 (675.2-1700.1)	943.0 (674.8-1671.5)	1231.7 (685.4-1782.5)	0.799

K18 (U/L)	964.7 (264.6-1996.3)	665.6 (239.2-1748.8)	1198.5 (557.9-2475.6)	0.077

*P-values represent comparison of AD vs. ACLF calculated by t-test or Fisher's exact test when appropriate.

B.

Disease group	PRO-C4 (ng/mL)	PRO-C5 (ng/mL)
Healthy controls (n=30)	161.0 (111.2-187.3)	321.0 (198.2-403.0)
Stable cirrhosis (n=39)	200.5 (158.5-249.8)	534.3 (377.1-845.2)
AD+ACLF (n=144)	228.7 (173.3-294.2)	539.9 (368.5-706.5)
**P-value***	**<0.001**	**0.004**
AD (n=116)	231.6 (170.2-296.7)	538.9 (367.2-705.4)
ACLF (n=28)	226.2 (182.3-288.3)	543.8 (380.9-824.8)
**P-value**	0.837	0.149
AD (n=116)	231.6 (170.2-296.7)	538.9 (367.2-705.4)
ACLF 1 (n=16)	219.8 (171.1-254.9)	543.8 (419.1-666.4)
ACLF 2 (n=9)	279.3 (179.3-310.6)	706.1 (393.5-856.4)
ACLF 3 (n=3)	192.9 (178.8-192.9)	408.3 (352.8-638.4)
**P-value** (ACLF grade 1 vs. 2+3)**	0.872	0.610

* One-way ANOVA or Welch's test over the three groups.
**Two-sample's t-test between ACLF grade 1 vs. grade 2+3.

Table S3. Spearman rank-order correlation analysis for PRO-C8 (A) and ARGS (B), C4M (C), C6M (D), PRO-C4 (E), PRO-C5 (F) with markers of organ injury, inflammation, cell death and prognosis in the study cohorts.

A.

**Variables**	**Spearman *r* for PRO-C8**	**P-value**
**Liver and kidney injury**		
Bilirubin	0.130	0.163
Albumin	0.024	0.808
INR	0.100	0.287
Creatinine	-0.220	**0.017**
NGAL	-0.143	0.141
**Inflammation**		
WCC	0.115	0.217
IL-6	-0.019	0.859
IL-8	0.235	**0.029**
IL-10	-0.185	0.089
IL-1RA	0.016	0.882
sCD163	0.080	0.373
**Cell death**		
cK18	-0.055	0.722
K18	-0.203	0.196
**Prognostic scores**		
MELD score	0.006	0.949
CLIF-C AD score*	0.017	0.879
CLIF-C ACLF score**	-0.003	0.989
CLIF-C OF score	-0.033	0.733

B.

**Variables**	**Spearman *r* for ARGS**	**P-value**
**Liver and kidney injury**		
Bilirubin	0.124	0.180
Albumin	-0.126	0.202
INR	0.115	0.218
Creatinine	-0.074	0.425
NGAL	0.098	0.313
**Inflammation**		
WCC	0.057	0.539
IL-6	0.162	0.138
IL-8	0.030	0.785
IL-10	0.007	0.947
IL-1RA	-0.087	0.426
sCD163	0.055	0.539
**Cell death**		
cK18	0.200	0.193
K18	0.106	0.502
**Prognostic scores**		
MELD score	0.103	0.268
CLIF-C AD score*	-0.193	0.079
CLIF-C ACLF score**	0.131	0.497
CLIF-C OF score	0.179	0.059

C.

Variables	Spearman *r* for C4M	P-value
**Liver and kidney injury**		
Bilirubin	0.222	**0.015**
Albumin	-0.075	0.440
INR	0.113	0.222
Creatinine	-0.144	0.118
NGAL	-0.117	0.228
**Inflammation**		
WCC	0.092	0.321
IL-6	-0.168	0.120
IL-8	0.286	**0.007**
IL-10	-0.064	0.557
IL-1RA	-0.090	0.405
sCD163	0.121	0.175
**Cell death**		
cK18	0.006	0.971
K18	-0.152	0.342
**Prognostic scores**		
MELD score	0.077	0.407
CLIF-C AD score*	-0.029	0.791
CLIF-C ACLF score**	-0.162	0.411
CLIF-C OF score	0.073	0.445

D.

Variables	Spearman *r* for C6M	P-value
**Liver and kidney injury**		
Bilirubin	0.247	**0.007**
Albumin	-0.096	0.324
INR	0.169	0.067
Creatinine	-0.116	0.209
NGAL	-0.019	0.848
**Inflammation**		
WCC	0.186	0.044
IL-6	-0.072	0.510
IL-8	0.370	**<0.001**
IL-10	-0.029	0.789
IL-1RA	0.033	0.762
sCD163	0.179	**0.043**
**Cell death**		
cK18	-0.137	0.375
K18	-0.271	0.083
**Prognostic scores**		
MELD score	0.140	0.131
CLIF-C AD score*	0.114	0.294
CLIF-C ACLF score**	0.025	0.899
CLIF-C OF score	0.069	0.469

E.

Variables	Spearman *r* for PRO-C4	P-value
**Liver and kidney injury**		
Bilirubin	0.171	**0.047**
Albumin	-0.064	0.533
INR	-0.050	0.565
Creatinine	-0.067	0.440
NGAL	0.031	0.736
**Inflammation**		
WCC	0.027	0.756
IL-6	-0.139	0.214
IL-8	0.082	0.463
IL-10	-0.149	0.181
IL-1RA	-0.111	0.317
sCD163	0.139	0.100
**Cell death**		
cK18	0.238	0.086
K18	0.087	0.543
**Prognostic scores**		
MELD score	0.106	0.222
CLIF-C AD score*	-0.148	0.130
CLIF-C ACLF score**	-0.100	0.667
CLIF-C OF score	-0.131	0.191

F.

Variables	Spearman *r* for PRO-C5	P-value
**Liver and kidney injury**		
Bilirubin	0.161	0.066
Albumin	-0.020	0.848
INR	-0.041	0.640
Creatinine	-0.120	0.169
NGAL	0.003	0.973
**Inflammation**		
WCC	0.169	0.054
IL-6	0.035	0.760
IL-8	0.182	0.105
IL-10	-0.083	0.467
IL-1RA	0.119	0.293
sCD163	0.070	0.414
**Cell death**		
cK18	0.103	0.466
K18	0.065	0.655
**Prognostic scores**		
MELD score	0.066	0.453
CLIF-C AD score*	0.020	0.841
CLIF-C ACLF score**	-0.199	0.388
CLIF-C OF score	-0.081	0.424

* This analysis could be performed in AD patients only (n=104)
**This analysis could be performed in ACLF patients only (n=35).

Table S4. Multivariate competing-risk regression analysis for 28- and 90-day mortality in the study cohort A (n=139).

	**Mortality at 28 days**		**Mortality at 90 days**	
	**HR (95% CI)**	**p-value**	**HR (95% CI)**	**p-value**
**All patients (n=139)**	15 died, 4 transplanted		29 died, 11 transplanted	
Log(PRO-C6)<br>CLIF-C OF score	1.864 (0.436-7.971)<br>1.670 (1.272-2.194)	0.401<br>**<0.001**	1.755 (0.694-4.434)<br>1.529 (1.239-1.888)	0.234<br>**<0.001**
**ACLF patients (n=35)**	10 died, 2 transplanted		14 died, 6 transplanted	
Log(PRO-C6)<br>CLIF-C ACLF score	1.139 (0.129-10.03)<br>1.136 (1.024-1.260)	0.907<br>**0.016**	0.503 (0.108-2.337)<br>1.202 (1.081-1.337)	0.381<br>**<0.001**

Table S5. CPE of PRO-C3 and PRO-C6 for 28- and 90-day mortality in association with those of the CLIF-C OF and CLIF-C ACLF score in the study cohort A.

	**Mortality at 28 days**	**Mortality at 90 days**
	**CPE (95% CI)**	**CPE (95% CI)**
**All patients (n=139)**		
PRO-C3	0.562 (0.410-0.715)	0.552 (0.445-0.659)
PRO-C6	0.704 (0.625-0.783)	0.653 (0.578-0.728)
CLIF-C OF score	0.698 (0.642-0.753)	0.680 (0.629-0.730)
CLIF-C OF score + PRO-C3	0.717 (0.655-0.779)	0.690 (0.633-0.747)
CLIF-C OF score + PRO-C6	0.726 (0.665-0.787)	0.702 (0.648-0.756)
**ACLF patients (n=35)**		
PRO-C3	0.549 (0.384-0.715)	0.523 (0.359-0.687)
PRO-C6	0.636 (0.520-0.752)	0.596 (0.478-0.714)
CLIF-C ACLF score*	0.699 (0.592-0.806)	0.728 (0.635-0.820)
CLIF-C ACLF score + PRO-C3*	0.770 (0.624-0.915)	0.838 (0.753-0.923)
CLIF-C ACLF score + PRO-C6*	0.699 (0.583-0.815)	0.751 (0.652-0.851)

Table S6. Baseline characteristics of the 25 ACLF patients included in the validation cohort compared to the 35 ACLF patients included in the study cohort A.

**Variable**	**ACLF patients**		**P-value**
	**CANONIC (n=35)**	**NIS (n=25)**	
**Age, mean**	54.9 ± 10.8	46.8 ± 10.5	**0.006**
**Gender (male), n (%)**	23 (65.7)	14 (56)	0.591
**Laboratory parameters**			
Bilirubin (umol/L)	207.8 (46.6-365.1)	282 (130-492)	0.090
Albumin (g/L)	28.4 ± 5.1	31.1 ± 5.7	0.080
INR	2.1 (1.5-2.5)	2.1 (1.8-2.4)	0.679
Creatinine (umol/L)	185.2 (100.8-257.9)	104.0 (77.0-205.0)	**0.033**
WCC ($x10^9$/L)	6.6 (4.6-10.2)	11.2 (7.2-15.1)	**0.002**
**ACLF grade (I/II/III), n (%)**	19/12/4 (54.3/34.3/11.4)	9/8/8 (36/32/32)	0.130
**CLIF-C OF score,**	10 (9-11)	11 (10-13)	**<0.0001**
**CLIF-C ACLF score**	46.5 (42-50)	52.1 (45.9-61.1)	**0.004**
**Organ Failures, n (%)**			
Liver	19 (54.3)	15 (60)	0.793
Kidney	18 (51.4)	13 (52)	1
Brain	4 (11.4)	4 (16)	0.708
Respiratory	1 (2.9)	7 (28)	**0.007**
Circulatory	6 (17.1)	9 (36)	0.133
Coagulation	9 (25.7)	6 (24)	1
**Mortality, n (%)**			
28-days	10 (28.6)	12 (48)	0.175
90-days	14 (40)	16 (64)	0.115
**Transplantation, n (%)**			
28-days	2 (6)	0	n.a.
90-days	6 (17)	0	n.a.

*P-values represent comparison of CANONIC vs. NIS calculated by t-test or Fisher's exact test when appropriate.

Table S7. Multivariate Cox regression analysis for 28- (A) and 90-day (B) mortality in the validation cohort of 25 patients admitted with ACLF.

A.

Variable	HR (95% CI)	P-value
**Day 1 data**		
Log(PRO-C6)<br>CLIF-C OF score	2.720 (0.635-11.645)<br>1.350 (1.039-1.756)	0.178<br>**0.025**
Log(PRO-C6)<br>CLIF-C ACLF score	2.533 (0.592-10.836)<br>1.089 (1.012-1.172)	0.210<br>**0.024**
**Day 4 data**		
Log(PRO-C6)<br>CLIF-C OF score	6.249 (0.225-173.363)<br>1.230 (0.833-1.816)	0.280<br>0.298
Log(PRO-C6)<br>CLIF-C ACLF score	5.881 (0.323-107.110)<br>1.061 (0.968-1.163)	0.231<br>0.207
**Day 7 data**		
Log(PRO-C6)<br>CLIF-C OF score	1.045 (0.207-5.272)<br>1.662 (1.181-2.339)	0.958<br>**0.004**
Log(PRO-C6)<br>CLIF-C ACLF score	2.245 (0.526-9.581)<br>1.097 (1.025-1.173)	0.274<br>**0.007**

B.

Variable	HR (95% CI)	P-value
**Day 1 data**		
Log(PRO-C6)<br>CLIF-C OF score	2.389 (0.668-8.544)<br>1.414 (1.123-1.779)	0.180<br>**0.003**
Log(PRO-C6)<br>CLIF-C ACLF score	2.019 (0.546-7.466)<br>1.113 (1.040-1.191)	0.292<br>**0.002**
**Day 4 data**		
Log(PRO-C6)<br>CLIF-C OF score	25.883 (1.526-439.139)<br>1.027 (0.771-1.368)	**0.024**<br>0.857
Log(PRO-C6)<br>CLIF-C ACLF score	13.823 (0.96-199.013)<br>1.028 (0.949-1.114)	0.054<br>0.492
**Day 7 data**		
Log(PRO-C6)<br>CLIF-C OF score	1.444 (0.313-6.666)<br>1.692 (1.194-2.399)	0.638<br>**0.003**
Log(PRO-C6)<br>CLIF-C ACLF score	3.206 (0.801-12.829)<br>1.097 (1.025-1.174)	0.100<br>**0.008**

Table S8. CPE of PRO-C6 at day 1, day 4 and day 7 for 28- (A) and 90-day (B) mortality in 25 patients with ACLF in the validation cohort.

A.

Variable	CPE (95% CI)
**Day 1 data**	
Log(PRO-C3)	0.515 (0.354-0.677)
Log(PRO-C6)	0.667 (0.538-0.796)
CLIF-C OF score	0.686 (0.586-0.786)
CLIF-C ACLF score	0.731 (0.618-0.845)
**Day 4 data**	
Log(PRO-C3)	0.579 (0.383-0.774)
Log(PRO-C6)	0.773 (0.652-0.895)
CLIF-C OF score	0.739 (0.625-0.854)
CLIF-C ACLF score	0.738 (0.618-0.857)
**Day 7 data**	
Log(PRO-C3)	0.572 (0.396-0.748)
Log(PRO-C6)	0.751 (0.626-0.877)
CLIF-C OF score	0.825 (0.736-0.913)
CLIF-C ACLF score	0.817 (0.721-0.912)
**Delta day 1-4**	
Log(PRO-C6)	0.685 (0.540-0.830)
**Delta day 1-7**	
Log(PRO-C6)	0.587 .448-0.726)

B.

Variable	CPE (95% CI)
**Day 1 data**	
Log(PRO-C3)	0.552 (0.413-0.690)
Log(PRO-C6)	0.660 (0.542-0.777)
CLIF-C OF score	0.700 (0.616-0.784)
CLIF-C ACLF score	0.759 (0.670-0.848)
**Day 4 data**	
Log(PRO-C3)	0.610 (0.465-0.755)
Log(PRO-C6)	0.781 (0.677-0.885)
CLIF-C OF score	0.711 (0.600-0.822)
CLIF-C ACLF score	0.721 (0.610-0.833)
**Day 7 data**	
Log(PRO-C3)	0.581 (0.431-0.731)
Log(PRO-C6)	0.770 (0.665-0.875)
CLIF-C OF score	0.835 (0.754-0.917)
CLIF-C ACLF score	0.821 (0.731-0.912)
**Delta day 1-4**	
Log(PRO-C6)	0.698 (0.584-0.812)
**Delta day 1-7**	
Log(PRO-C6)	0.612 (0.502-0.723)

SUPPLEMENTARY REFERENCE

1. Schneider CA, Rasband WS, Eliceiri KW. NIH Image to ImageJ: 25 years of image analysis. Nat Methods 2012;9:671-675.
