# Supplementary material for: Biomarkers of extracellular matrix formation are associated with acute-on-chronic liver failure
Source: JHEP Rep. 2021 Aug 27;3(6):100355. doi: 10.1016/j.jhepr.2021.100355 (PMC8581571; doi:10.1016/j.jhepr.2021.100355)
Supplement: Multimedia component 2 [file mmc2.pdf]

JOURNAL
OF HEPATOLOGY

Journal of Hepatology

CTAT methods

Tables for a "Complete, Transparent, Accurate and Timely account" (CTAT) are now mandatory for all revised submissions. The aim is to enhance the reproducibility of methods.

- Only include the parts relevant to your study
- Refer to the CTAT in the main text as 'Supplementary CTAT Table'
- Do not add subheadings
- Add as many rows as needed to include all information
- Only include one item per row

If the CTAT form is not relevant to your study, please outline the reasons why:

1.1 Antibodies

Name	Citation	Supplier	Cat no.	Clone no.
Smooth Muscle Actin		Agilent Dako	M085101-2	1A4

1.2 Cell lines

Name	Citation	Supplier	Cat no.	Passage no.	Authentication test method

1.3 Organisms

Name	Citation	Supplier	Strain	Sex	Age	Overall n number

1.4 Sequence based reagents

Name	Sequence	Supplier

1.5 Biological samples

Description	Source	Identifier
Study Cohort A (plasma samples)	HCB-IDIBAPS Biobank in Barcelona, Centre Esther Koplowitz (CEK, Rosselló 153) 08036 Barcelona, Spain	CANONIC trial
Study Cohort B (plasma samples)	HCB-IDIBAPS Biobank in Barcelona, Centre Esther Koplowitz (CEK, Rosselló 153) 08036 Barcelona, Spain	CANONIC trial
Healthy Volunteers (plasma samples)	Nordic Biosciences, Herlev Hovedgade 205, 207, 2730 Herlev, Denmark	n.a.
Stable Cirrhosis patients (plasma samples)	Institute for Liver and Digestive Health, University College London, Royal Free Campus, London; United Kingdom	n.a.
Validation cohort (plasma samples)	Institute for Liver and Digestive Health, University College London, Royal Free Campus, London; United Kingdom	Non-Interventional Study (NIS)
Alcoholic liver cirrhosis patients (liver biopsies)	Sheila Sherlock Liver Centre, Royal Free London NHS Foundation Trust, London, UK	n.a.

1.6 Deposited data

Name of repository	Identifier	Link

1.7 Software

Software name	Manufacturer	Version
R Statistical Software	R Core Team	4.0.4
Biorender.com	Biorender	n.a.
ImageJ	ImageJ	1.53a

1.8 Other (e.g. drugs, proteins, vectors etc.)

ELISA	Cat. No.	Lot. No.
PRO-C3	1700-05/06	EB1809A and EB1911A
PRO-C4	8000	MJ1806A
PRO-C5	3000	SE1708B
PRO-C6	4000-02	BL1810A and BL1908A
PRO-C8	R1019-00	COH1806A
C4M	1300-01	MU1807A
C6M	1500-01	TB1809A
ARGS	4900	HER1810A

1.9 Please provide the details of the corresponding methods author for the manuscript:

Prof. Rajiv Jalan
E-mail: r.jalan@ucl.ac.uk

2.0 Please confirm for randomised controlled trials all versions of the clinical protocol are included in the submission. These will be published online as supplementary information.
